# Pathogenicity and pathobiological characterization of a recombinant genotype I/II African swine fever virus in pigs

**DOI:** 10.1080/21505594.2025.2580123

**Published:** 2025-10-25

**Authors:** Hu Suk Lee, Byungkwan Oh, Vuong Nghia Bui, Duy Tung Dao, Ngoc Anh Bui, Su-Beom Chae, Minh Duc Nguyen Do, Mai Trang Tran, Quy Duy Nguyen, Young-Sik Kim, Jeong Ah Park, Seong-Keun Hong, Ki-Hyun Cho, Yeon-Hee Kim, Jae-Ku Oem, Bumseok Kim, Chang-Gi Jeong, Sang-Ik Oh

**Affiliations:** a College of Veterinary Medicine, Chungnam National University, Daejeon, Republic of Korea; b Laboratory of Veterinary Pathology and Biosafety Research Institute, College of Veterinary Medicine, Jeonbuk National University, Iksan, Republic of Korea; c Virology Department, National Institute of Veterinary Research, Hanoi, Vietnam; d Laboratory of Veterinary Infectious Disease and Biosafety Research Institute, College of Veterinary Medicine, Jeonbuk National University, Iksan, Republic of Korea; e Foreign Animal Disease Division, Animal and Plant Quarantine Agency, Gimcheon, Republic of Korea

**Keywords:** African swine fever virus, recombinant genotypes I and II, pathogenicity, pigs, Vietnam

## Abstract

African swine fever virus (ASFV) is a highly contagious pathogen affecting domestic pigs and wild boar, causing substantial economic losses. Recently, a highly virulent recombinant ASFV strain combining genotypes I and II (rASFV I/II) emerged in Asia. Genotype II ASFV vaccine candidates have failed to effectively protect against rASFV I/II infection, presenting a critical challenge. Here, we investigated the pathobiological characteristics of rASFV I/II in domestic pigs. Ten healthy 7-week-old female pigs were intramuscularly inoculated with an rASFV I/II strain. Clinical signs, rectal temperatures, and samples were collected daily, and necropsies were conducted postmortem. All inoculated pigs succumbed to infection within 5–7 days post-inoculation (dpi), with a mean of 5.5 ± 0.7 dpi. High fever ( > 40.5°C) was observed at an average onset of 2.6 ± 0.8 dpi, and the incubation period averaged 3.0 ± 1.1 dpi. Viral DNA was first detected in blood at 2 dpi, with viral genome copies increasing steadily. Postmortem analysis revealed severe congestive splenomegaly and hemorrhagic lymphadenopathy in all pigs. A preliminary comparison of histopathological lesions in early phase of viral infection showed that all three rASFV I/II-infected pigs showed endothelial injury lesions, while only one genotype II ASFV-infected pig showed this lesion. High viral DNA copy numbers were detected in all organs, particularly the liver, lymph nodes, spleen, lungs, and kidneys. These findings demonstrated distinct pathobiological features of rASFV I/II that warrant further investigation for their implications on disease control. The results also emphasized the urgent need for enhanced surveillance in Asia, rapid diagnostic methods, and effective vaccines targeting emerging ASFV variants.

## Introduction

African swine fever virus (ASFV) is an enveloped, large double-stranded DNA virus belonging to the family Asfarviridae [1,2]. ASFV infects members of the Suidae family – including domestic pigs, wild boar, warthogs, and bushpigs – as well as soft ticks (*Ornithodoros* spp.), and could cause a highly contagious hemorrhagic disease [3]. African swine fever (ASF) posed a major global threat to the swine industry, causing significant economic losses due to the virus’s high mortality rates ( > 90%) in domestic pigs [4].

Currently, 23 genotypes of ASFV have been identified, primarily in sub-Saharan Africa [5–7]. Only two genotypes have been detected outside Africa. Genotype I ASFV was first introduced from West Africa to Portugal between 1957 and 1960 [8]. In 2007, genotype II ASFV spread from the east coast of Africa to Georgia. In Asia, the first case of the Georgia07-like ASFV was detected in China in 2018 and quickly spread to neighboring Asian countries [9,10]. In Vietnam, the first ASF outbreak was reported in 2019 in Hung Yen province, approximately 50 km from Hanoi and 250 km from the Chinese border [11]. Le et al. (2019) identified the isolated strain as belonging to p72 genotype II, identical to the strains involved in the Chinese outbreaks [11]. Recently, a highly virulent recombinant ASFV strain combining genotypes I and II (referred to as “rASFV I/II”) has been detected in several Asian countries. In China, this strain was isolated in Jiangsu province (JS/LG/21), Henan province (HeN/123014/22), and Inner Mongolia (IM/DQDM/22) since 2021 [12]. In Vietnam, the rASFV I/II strain was detected in domestic pigs across six northern provinces in 2023 [13].

Several in vivo viral inoculation experiments have been conducted to evaluate the pathogenesis and clinical signs of genotype II ASFV-infected pigs in Vietnam [14,15]. Lee et al. (2021) reported that genotype II ASFV-infected pigs succumbed to infection between 5 and 8 days post-inoculation (dpi), with an incubation period of approximately 3.7 ± 0.5 days. Oh et al. (2023) estimated the basic reproduction number (R₀) to be 2.916 and 4.015 [14,15]. Recently, studies in Vietnam have assessed the characteristics of novel emerging rASFV I/II strains [13,16,17]. These studies primarily focused on the sequence variants of rASFV I/II or the protective efficacy of Vietnamese commercial ASF vaccines against rASFV I/II strains. Given the emergence of rASFV I/II strains in several Asian countries, including China, Vietnam, and Russia [12,13,18], understanding the pathobiological characteristics of this ASFV variant and the associated pathobiological responses in rASFV I/II-infected pigs is crucial for effective disease control and prevention. This study, therefore, aimed to investigate the clinical outcomes and pathogenicity of rASFV I/II strains in domestic pigs through in vivo viral inoculation experiments.

## Materials and methods

### rASFV I/II strain

The rASFV I/II strain used in this study was isolated in October 2023 from a pig’s blood sample collected during an ASF outbreak at a commercial farm in Hai Duong province, Vietnam. Sample collection was performed under the legal framework of mandatory ASF surveillance in Vietnam by the Vietnamese government institute (National Institute of Veterinary Research; NIVR), as ASF is a notifiable disease requiring immediate government intervention according to Vietnamese animal health regulations. No additional owner consent was needed for this official disease investigation. The virus was isolated using porcine alveolar macrophage (PAM) cells, and designated as “ASF/HD 231005.” Viral DNA was extracted using the Maxwell® RSC Whole Blood DNA Kit (Promega, USA) according to the manufacturer’s protocol. Approximately 500 ng of purified DNA was fragmented into 150–250 bp segments using the Bioruptor Pico sonication system (Diagenode, Belgium) for library preparation. The fragmented DNA libraries were hybridized with ASFV-specific biotinylated capture probes (Celemics, Korea). Target-enriched fragments were isolated using streptavidin-coated paramagnetic beads, and unbound DNA was removed by magnetic separation. Captured DNA was eluted through enzymatic digestion of the cRNA capture probes, followed by purification. The enriched libraries were subsequently sequenced on the NextSeq 550 platform (Illumina, USA) using 2 × 151 bp paired-end reads. The raw sequencing reads were subjected to adapter and quality trimming by Trimmomatic v0.39 program. The quality of trimmed datasets was evaluated using FastQC v0.11.9 to ensure removal of low-quality bases and sequencing artifacts. De novo assembly of trimmed sequences was performed using SPAdes genome assembler v4.0.0. Regions containing assembly gaps or ambiguous bases were targeted by PCR amplification using gene-specific primers (GSPs) designed from adjacent contigs. The resulting amplicons were sequenced using Sanger sequencing (Macrogen, Korea), and the sequences were incorporated into the assembly to close gaps and resolve low-confidence regions. The finalized whole-genome sequence was queried against the NCBI database using BLASTn to identify the most closely related ASFV strain. Genome annotation was performed using the Genome Annotation Transfer Utility (GATU), with the DQDM strain (rASFV I/II, Accession No. OQ504955) as the reference genome. The whole-genome sequence of ASF/HD 231005 has been deposited in GenBank under accession number PX212717 (https://www.ncbi.nlm.nih.gov/nuccore/PX212717). In addition, the full nucleotide sequence of three major ASFV structural genes, including *B646L* (p72), *E183L* (p54), and *EP402R* (CD2v), of the ASF/HD 231005 strain has been deposited in NCBI GenBank under the accession numbers PQ328515 (https://www.ncbi.nlm.nih.gov/nuccore/PQ328515), PV614732 (https://www.ncbi.nlm.nih.gov/nuccore/PV614732), and PV614731 (https://www.ncbi.nlm.nih.gov/nuccore/PV614731), respectively.

### Phylogenetic analysis of rASFV I/II strain

To determine the genotypes (*B646L* and *E183L*) and serogroup (*EP402R*) of the ASF/HD 231005 strain, reference nucleotide sequences of the *B646L*, *E183L*, and *EP402R* genes were retrieved from the GenBank database. Multiple sequence alignments were performed using Clustal Omega v1.2.4 with default parameters. The best-fit models for each gene were identified using the ModelFinder module within IQ-TREE v2.2.0. Maximum likelihood phylogenetic trees were constructed using IQ-TREE, with 1,000 ultrafast bootstrap replicates to assess branch support. Tree visualization and annotation were conducted in R v4.3.0 (R Core Team, Vienna, Austria) using the ggtree and ggplot2 packages.

### Animal experiment

A total of 15 healthy female pigs (Yorkshire × Landrace × Duroc), aged 7 weeks, were obtained from the same herd at a commercial pig farm in Vietnam. We determined our sample size based on our previous studies [14,15], and selected only female pigs to minimize sex-based physiological differences. All piglets were vaccinated against porcine circovirus 2 and classical swine fever virus before entering the biosafety and laboratory facilities at the NIVR, Vietnam. The pigs were screened for major endemic diseases in Vietnam and thoroughly inspected by veterinary researchers before introduction into the laboratory facility. Upon arrival, the pigs were confirmed seronegative for foot-and-mouth disease virus, porcine reproductive and respiratory syndrome virus, and ASFV before inoculation with the rASFV I/II strain. The pigs were fed a commercial diet twice daily and provided water *ad libitum*. Room temperature and humidity in the biosafety facility were monitored and recorded daily.

Fifteen pigs were randomly divided by veterinarians in NIVR into two groups: the rASFV I/II strain inoculation group (rVI, *n* = 10) and the negative control group (NC, *n* = 5). The number of pigs was determined based on the sample size used in our previous study on genotype II ASFV [14], with survival rate as the primary outcome measure for comparison of results. To ensure balanced allocation, pigs were stratified based on visible body size prior to randomization. To minimize potential confounders, the experimental pigs were housed in separate rooms with controlled environmental conditions, preventing cross-contamination and ensuring group-specific outcomes. Pigs in the rVI group were housed in an enhanced animal biosafety level 2 facility and intramuscularly inoculated with 1 mL of the rASFV I/II strain at a dose of 10^3.5^ 50% hemadsorbing dose (HAD_50_/mL). The five pigs in the NC group were inoculated with sterile phosphate-buffered saline (PBS) and housed under standard conditions. During the experiment, the room temperature ranged from 25–26°C, and the relative humidity ranged from 60–80% for both animal housing facilities.

This first animal experiment (rASFV I/II inoculation) was conducted from June to July 2024. Fifteen pigs arrived at the NIVR facility on 24 June 2024, and were acclimatized for 7 days. On 1 July 2024, pigs in the rVI group were inoculated with rASFV I/II, while control pigs received PBS. The experiment concluded on 8 July 2024, when the last infected pig succumbed to infection. A detailed protocol, including the research question, experimental design, and analysis plan, was prepared prior to the study. However, the protocol was not registered in a public repository due to institutional guidelines. All experimental animals and data were included in the analysis.

### Clinical assessment, sample collection, and gross lesions of rASFV I/II-infected pigs

The daily clinical signs and rectal temperatures of all pigs (*n* = 15) were recorded throughout the experiment. Clinical sign scores (CS) were calculated following inoculation, and samples were collected as described in a previous study [14]. Briefly, total CS > 3 indicated the presence of clinical signs associated with ASFV infection. Accordingly, the incubation period was defined as the number of days from inoculation to the first day a pig’s total CS > 3. Blood samples and three types of swab samples (oral, nasal, and rectal) were collected daily from all experimental pigs. To minimize the pain and distress of pigs, all sample collection procedures were conducted gently and aseptically by trained veterinarians. Environmental swab samples, including pooled feces from the floor, feed, and water, were also obtained daily. In addition, herd-based oral fluid samples were collected using ropes daily until 4 dpi, following the protocol of our previous study [14]. Humane endpoints were determined according to our previous study [19]. In detail, animals that reached total CS > 10 and exhibited severe tremors or immobility (i.e., shivering and inability to move) were considered to have met the predetermined humane endpoints.

Necropsies were performed immediately after death by a designated veterinary pathologist, who was blinded to the group allocations. The pathologist documented gross lesions primarily in 11 organ tissues: spleen, submandibular lymph node (SLN), gastrohepatic lymph node (GLN), mesenteric lymph node (MLN), tonsils, lungs, liver, kidneys, heart, brain, and spinal cord. These tissues were also collected for the quantification of ASFV genome copies. In addition, spleen and liver tissues from all pigs were fixed in 10% neutral buffered formalin, and embedded in paraffin. Five-μm sections were stained with hematoxylin and eosin (H&E) to evaluate histopathological lesions.

### Viral gene (p72) detection by quantitative polymerase chain reaction (qPCR) analysis and enzyme-linked immunosorbent assay (ELISA) for serum samples

Viral DNA was extracted from all collected samples [blood, three types of swab samples, environmental samples, oral fluids, and 11 organ tissues from deceased pigs (*n* = 15)] using the WizPrep™ Viral DNA/RNA Mini Kit (V2) (Wizbiosolutions INC., Seongnam, Korea), following the manufacturer’s instructions. The extracted DNA was analyzed for ASFV DNA presence using a VDx ASFV qPCR kit (Median Diagnostics, Cat. No. NS-ASF-31, Chuncheon, Korea), as described in our previous study [14]. Viral copy numbers were determined by using a standard curve generated from serial dilutions of the quantified ASFV DNA standard which was provided by the manufacturer of qPCR kit.

All serum samples collected during the entire experimental period were tested using the commercial ID Screen® African swine fever Indirect ELISA (Idvet, Grabels, France), which detects ASFV antibodies against three viral proteins (p32, p62, and p72).

### Comparison of histopathological lesions between genotype II ASFV- and rASFV I/II-inoculated pigs at 3 dpi

To partially address the direct comparison between genotype II ASFV and rASFV I/II infection in pigs, we conducted a preliminary comparative animal experiment from December 2024 to January 2025. Seven healthy pigs (aged 7 weeks) arrived at the NIVR facility on 23 December 2024, and were acclimatized for 7 days under identical conditions as described above. The pigs were randomly divided into two groups for rASFV I/II-inoculated (*n* = 3) and genotype II ASFV-inoculated group (*n* = 3). An additional pig served as a negative control and was inoculated with sterile PBS. The genotype II ASFV-inoculated group was intramuscularly inoculated with 1 mL of virus (GenBank accession no. OP615344) at a dose of 10^3.5^ HAD₅₀/mL, as previously described [15]. The rASFV I/II-inoculated group was inoculated with the same dose of ASF/HD 231005 strain used in the main experiment. All groups were inoculated simultaneously on 30 December 2024. All pigs were monitored for clinical signs and rectal temperatures as described above. At 3 dpi (2 January 2025), all pigs were humanely euthanized and immediately necropsied by trained veterinary pathologists. Anesthesia and euthanasia were performed in accordance with the American Veterinary Medical Association (AVMA) Guidelines for the Euthanasia of Animals (2020) and institutional veterinary policies. The pigs were intramuscularly premedicated with ketamine hydrochloride (20 mg/kg, Yuhan Ketamine 50, Yuhan Corp., Seoul, Republic of Korea) for sedation. Once adequately sedated, euthanasia was performed using a penetrating captive bolt applied to the frontal region of the skull, followed immediately by exsanguination via transection of the axillary vessels to ensure death. A total of five organ tissues – spleen, SLN, lungs, liver, and kidneys – were collected and fixed in 10% neutral buffered formalin. Formalin-fixed tissues were transported from Vietnam to Korea for histopathological processing. Due to international quarantine requirements, histopathological examination was performed from August 1 to 16, 2025. Following standard histological processing, tissues were embedded in paraffin, sectioned at 5 μm, and stained with hematoxylin and eosin (H&E). Histopathological lesions were evaluated by veterinary pathologists blinded to the group allocation. Lesion severity was graded as: 0 (no lesions), 1 (mild), 2 (moderate), or 3 (severe), following the scoring system described in our previous study [20] with minor modifications regarding the early phase of viral infection. The mean lesion scores for each organ were calculated and compared between groups.

### Statistical analysis

Survival rates were analyzed using the Kaplan–Meier method, and group differences were evaluated with the log-rank test. A linear mixed-effects model for repeated measures was applied to evaluate the time-course determinants of rectal temperature and CS in relation to experimental groups. When the group × time interaction in the mixed model was significant, Student’s t-tests were performed to compare the mean values of rectal temperature and CS between the two groups at the same time point. In addition, a one-way ANOVA was used to assess potential differences in viral loads across various organ tissue samples from rVI group pigs. Data for the two experimental groups are presented as the mean ± standard deviation, calculated based on daily measurements from individual pigs. Statistical analyses were conducted using SPSS version 29.0 (IBM, Armonk, NY, USA). Results with *p* < 0.05 were considered statistically significant.

## Results

### Phylogenetic analysis of the inoculation virus

**Figure 1.**
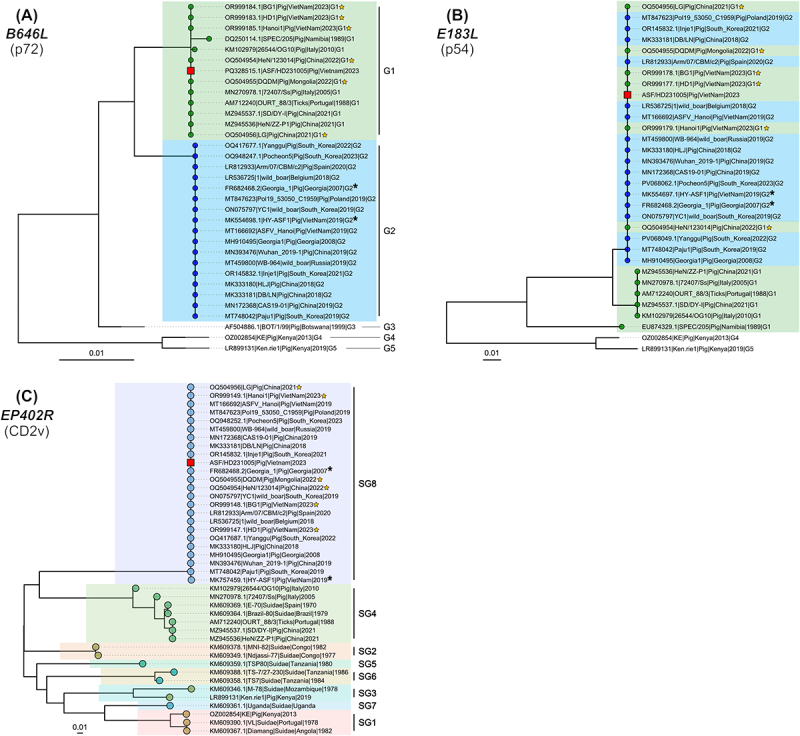
Phylogenetic analysis of African swine fever virus (ASFV) based on the nucleotide sequences of (A) *B646L* (p72), (B) *E183L* (p54), and (C) *EP402R* (CD2v) genes. Strains used in this study are marked with red squares, while previously reported recombinant ASFV (rASFV I/II) strains are indicated with yellow stars. Asterisks (*) represent the first reported genotype II ASFV in each country. Genotypes (G) were assigned based on *B646L* (p72) sequences, and serogroups (SG) were determined according to *EP402R* (CD2v) phylogeny. Scale bars display the number of nucleotide substitutions per site (phylogenetic distance). Trees were constructed using the maximum likelihood method, with bootstrap values calculated from 1,000 replicates.

Phylogenetic analysis based on the *B646L* (p72) gene revealed that ASF/HD 231005 clustered within genotype I (G1), grouping closely with Vietnamese strains isolated in 2023 (Figure 1(A)). In contrast, the *E183L* (p54) gene of ASF/HD 231005 exhibited close phylogenetic relatedness to genotype II (G2) strains, including ASFV strains from Georgia, China, and South Korea (Figure 1(B)). In the *EP402R* (CD2v)-based phylogeny, ASF/HD 231005 was classified within serogroup 8 (SG8), clustering with rASFV I/II strains previously reported in China and Inner Mongolia (Figure 1(C)). These overall results indicated that ASF/HD 231005 possesses a recombinant genomic structure, comprising *B646L* from genotype I, *E183L* from genotype II, and a SG8-type *EP402R* gene. These findings support the classification of ASF/HD 231005, which was used in this study, as a rASFV I/II strain.

### Pathobiological responses in rASFV I/II-inoculated pigs

**Figure 2.**
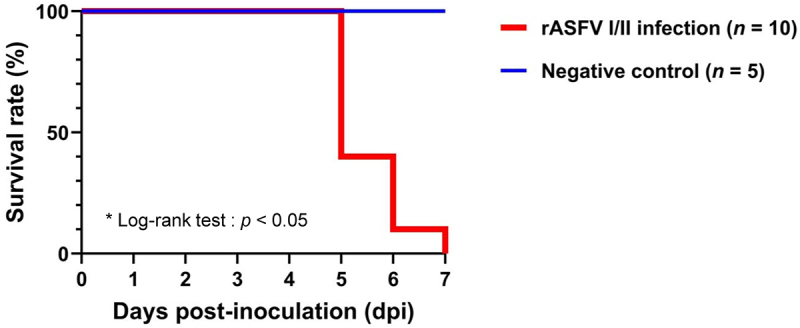
Survival rates (%) in the genotype I and II recombinant ASFV (rASFV I/II) infection group (red line) and negative control group (blue line).

**Figure 3.**
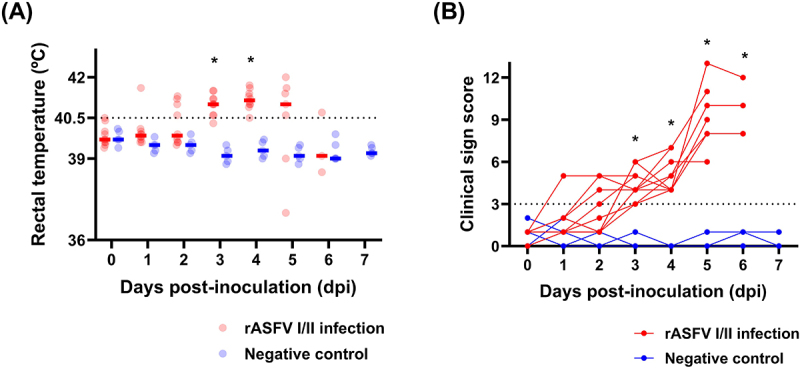
(A) Individual rectal temperatures of pigs from the rASFV I/II infection and negative control groups. Red and blue circles represent individual temperatures, while horizontal lines indicate the mean temperatures for the ASFV infection and negative control groups, respectively. Red and blue short lines represent the average temperature for the ASFV infection and negative control groups at each time point. *p < 0.05. (B) Clinical scores of pigs from the rASFV I/II infection group (red lines) and negative control group (blue lines). The scores were calculated based on the protocol published by Lee et al. [14]. *p < 0.05.

All pigs in the rVI group succumbed to infection within 5–7 dpi (average 5.5 ± 0.7 dpi). Notably, 6 of 10 rVI pigs (60%) died at 5 dpi (Figure 2). Kaplan–Meier analysis confirmed a significantly (χ^2^ = 11.3, *p* < 0.01) lower survival rate in the infected group. One pig (no. #32) was euthanized, because the pig reached our predetermined humane endpoint. No unexpected adverse events were observed during the whole experimental period. The average onset of high fever ( > 40.5°C) in the rVI group was 2.6 ± 0.8 dpi (Figure 3(A)). One pig (pig no. #31) exhibited clinical signs of ASFV infection (score 5) as early as 1 dpi. The mean CS of the rVI group increased progressively as follows: 0.9 ± 0.3 (1 dpi), 1.7 ± 1.1 (2 dpi), 2.4 ± 1.5 (3 dpi), 4.4 ± 1.0 (4 dpi), 5.0 ± 1.1 (5 dpi), and 10.0 ± 1.4 (6 dpi) (Figure 3(B)). Based on the onset of clinical signs, the average incubation period for rASFV I/II-infected pigs was 3.0 ± 1.1 dpi.

### Viral excretion from rASFV I/II-infected pigs

**Figure 4.**
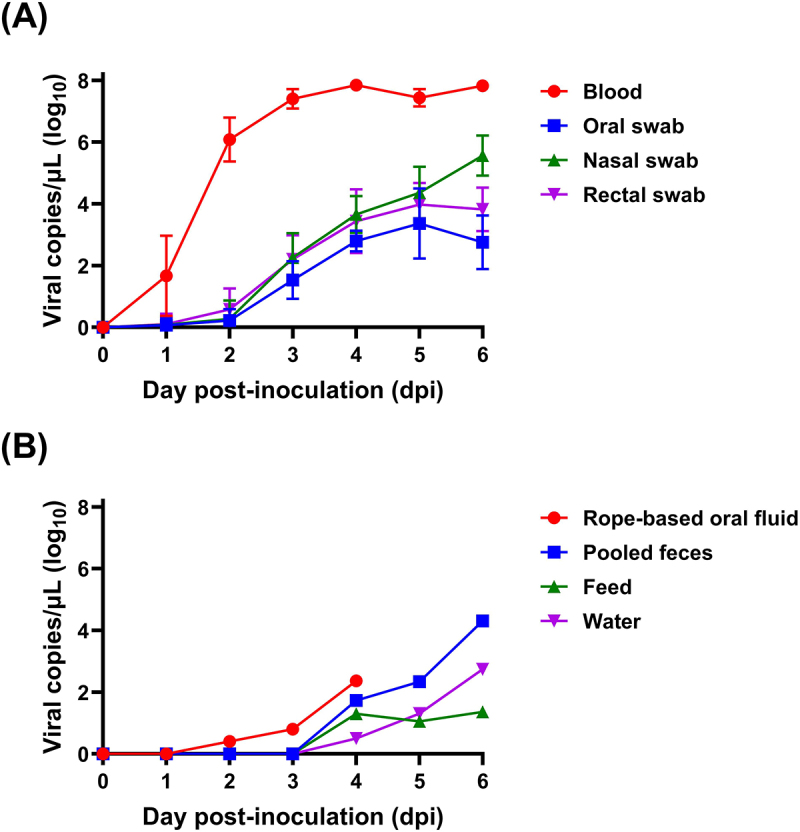
Time-serial mean copy numbers of African swine fever virus (ASFV) in the genotype I and II recombinant ASFV-infected pigs. (A) Individual samples: blood, oral swabs, nasal swabs, and rectal swabs. (B) Group samples: rope-based oral fluids, pooled feces, feed, and water samples.

All values are expressed as viral DNA copy numbers/μL (log₁₀). Additionally, all blood samples collected during the experiment tested seronegative for ASFV antibodies by ELISA. Seven pigs in the rVI group displayed viremia at 1 dpi, and viral genome DNA was detected in blood samples from all infected pigs by 2 dpi. The mean viral copy numbers in blood increased progressively from 1 to 4 dpi, with values of 1.67 ± 1.30 (1 dpi), 6.08 ± 0.72 (2 dpi), 7.41 ± 0.31 (3 dpi), and 7.85 ± 0.28 (4 dpi). Elevated viral loads persisted at 6 dpi (7.83 ± 0.28) (Figure 4(A)). Patterns of viral excretion in the rVI group showed a progressive increase in viral copy numbers across various swab types. In oral swabs, the mean viral copy numbers increased from 0.07 ± 0.23 at 1 dpi to a peak of 3.36 ± 1.14 at 5 dpi, followed by a slight decrease to 2.76 ± 0.87 at 6 dpi. Nasal swabs exhibited a steady increase in viral titers, rising from 0.09 ± 0.28 at 1 dpi to 5.56 ± 0.65 at 6 dpi. Similarly, rectal swabs showed an increase in viral loads from 0.11 ± 0.34 at 1 dpi to 3.82 ± 0.70 at 6 dpi, indicating sustained shedding through this route. Environmental samples tested positive for viral DNA starting at 4 dpi, with detected viral loads in pooled feces (1.73), feed (1.30), and water (0.20 log₁₀ copies/μL). Rope-based oral fluid samples tested positive as early as 2 dpi, with viral loads of 0.40 (Figure 4(B)).

### Gross and histopathological lesions in rASFV I/II-infected pigs

The major gross lesions observed in the rVI group pigs are summarized in Table 1. All rASFV I/II-infected pigs (*n* = 10, 100%) exhibited severe congestive splenomegaly and hemorrhagic enlargement of the MLN. Moderate-to-severe hemorrhagic lesions were present in the SLN and GLN in all rVI group pigs (*n* = 10, 100%). Hemorrhagic lesions were also observed in the lungs of all rVI group pigs, with severe lesions identified in seven pigs (70%). Although hyperemic lesions were noted in the brain of all rVI group pigs, most were mild (*n* = 7, 70%). Various degrees of hemorrhagic lesions were detected in the tonsils, heart, and kidneys. Severe ascites was observed in six pigs in the rVI group (60%). The representative histopathological lesions are shown in Fig. S1. All rASFV I/II-infected pigs showed severe congestive splenomegaly with moderate-to-severe lymphoid depletion and multifocal necrosis. The spleen tissue also showed numerous hemosiderin-laden macrophages. In addition, severe congestion lesions were also observed in liver tissue from rASFV I/II-infected pigs.

**Table 1.** Gross pathologic lesions from the genotype I and II recombinant ASFV (rASFV I/II)-infected pigs (*n* = 10).

Major pathological observation	Pig case no. (death period)										No. of pigs (%)			
	#4 (5 dpi)	#5 (5 dpi)	#6 (5 dpi)	#8 (6 dpi)	#12 (5 dpi)	#13 (6 dpi)	#15 (7 dpi)	#31 (5 dpi)	#32 (6 dpi)	#33 (5 dpi)	Intact(-)	Mild (+)	Moderate (++)	Severe (+++)
Skin erythema	–	–	–	–	–	–	–	+	+	–	8 (80%)	2 (20%)	0 (0%)	0 (0%)
Pericardial effusion	–	+	+++	+	–	–	++	+	+	–	4 (40%)	4 (40%)	1 (10%)	1 (10%)
Ascites or hemoperitoneum	+++	+++	++	+++	–	+++	–	+	+++	+++	2 (20%)	1 (10%)	1 (10%)	6 (60%)
Hemothorax	–	–	++	–	–	–	++	–	–	–	8 (80%)	0 (0%)	2 (20%)	0 (0%)
Pulmonary edema	–	–	–	–	–	–	+++	–	–	–	9 (90%)	0 (0%)	0 (0%)	1 (10%)
Congestive splenomegaly	+++	+++	+++	+++	+++	+++	+++	+++	+++	+++	0 (0%)	0 (0%)	0 (0%)	10 (100%)
Hemorrhagic enlargement in SLN	++	+++	++	+++	++	+++	+++	++	+++	+++	0 (0%)	0 (0%)	4 (40%)	6 (60%)
Hemorrhagic enlargement in GLN	++	+++	+++	+++	+++	+++	+++	++	+++	+++	0 (0%)	0 (0%)	2 (20%)	8 (80%)
Hemorrhagic enlargement in MLN	+++	+++	+++	+++	+++	+++	+++	+++	+++	+++	0 (0%)	0 (0%)	0 (0%)	10 (100%)
Hemorrhagic lesions in tonsil	++	+	+	+	–	–	+++	–	++	++	3 (30%)	3 (30%)	3 (30%)	1 (10%)
Hemorrhagic lesions in lung	+	+++	+++	+++	++	+++	+++	+++	++	+++	0 (0%)	1 (10%)	2 (20%)	7 (70%)
Hemorrhagic lesions in liver	–	–	–	–	–	–	–	–	–	–	10 (100%)	0 (0%)	0 (0%)	0 (0%)
Hemorrhagic lesions in kidney	–	++	+	++	–	++	+++	+++	+	++	2 (20%)	2 (20%)	4 (40%)	2 (20%)
Hemorrhagic lesions in heart	+	–	+++	–	+	–	–	+	–	+	5 (50%)	4 (40%)	0 (0%)	1 (10%)
Petechiae in intestine tracts	+	–	–	+	–	++	+	++	++	+++	3 (30%)	3 (30%)	3 (30%)	1 (10%)
Hyperemia of brain	++	+	+	+	+	++	+++	+	+	+	0 (0%)	7 (70%)	2 (20%)	1 (10%)
Hematochezia	–	–	–	–	–	++	–	–	–	–	9 (90%)	0 (0%)	1 (10%)	0 (0%)
Hemolytic jaundice	+++	+++	+++	+++	++	+++	+++	+++	+++	+++	0 (0%)	0 (0%)	1 (10%)	9 (90%)

*SLN: submandibular lymph node, GLN: gastrohepatic lymph node, MLN: mesenteric lymph node.

-: intact, +: mild, ++: moderate, +++: severe.

### Viral DNA detection in organ tissues from dead rVI group pigs

**Figure 5.**
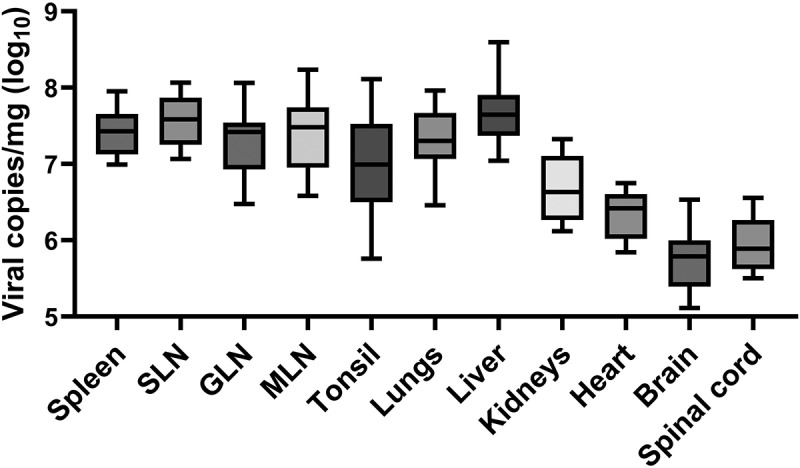
Viral genome copy numbers in 11 organ tissues—spleen, SLN (submandibular lymph node), GLN (gastrohepatic lymph node), MLN (mesenteric lymph node), tonsils, lungs, liver, kidneys, heart, brain, and spinal cord—collected from the genotypes I and II recombinant ASFV-infected pigs after postmortem examination.

Viral DNA was detected in all 11 collected organ tissues (spleen, SLN, GLN, MLN, tonsils, lungs, liver, kidneys, heart, brain, and spinal cord) from necropsied rVI group pigs (Figure 5). The mean viral DNA copy numbers were highest in the liver (7.67 ± 0.45), followed by the SLN (7.59 ± 0.33), spleen (7.42 ± 0.30), MLN (7.40 ± 0.53), lungs (7.34 ± 0.44), and GLN (7.29 ± 0.47). Viral DNA was also detected in other organ tissues, including the tonsils (7.01 ± 0.72), kidneys (6.67 ± 0.42), heart (6.34 ± 0.31), brain (5.76 ± 0.43), and spinal cord (5.95 ± 0.38). All values are expressed as viral DNA copy numbers/mg (log₁₀). A one-way ANOVA revealed no significant differences among the viral loads in these organs (*p* > 0.05).

### Direct comparison of histopathological lesions between genotype II ASFV and rASFV I/II at the early phase of infection

To directly compare the early pathological changes between genotype II ASFV and rASFV I/II-infected pigs, we examined histopathological lesions at 3 dpi (Fig. S2). The comparative lesion severity scores across five major organs are summarized in Table 2. The most notable differences were observed in lymphoid organs. In the spleen, while both groups showed severe congestion and erythrophagocytosis, necrosis with karyorrhexis was more frequent in the rASFV I/II group (2/3 pigs, with one showing moderate severity) compared to genotype II (1/3 pigs with mild severity). Moreover, the endothelial injury with microthrombi formation was already present in all rASFV I/II-infected pigs (3/3) but only in one genotype II ASFV-infected pig (1/3) at this early time point. This finding implied that rASFV I/II may cause more rapid and widespread vascular damage than genotype II ASFV infection. Similarly, in submandibular lymph node, the rASFV I/II group exhibited more severe necrosis (3/3 pigs with 2/3 showing moderate severity) than genotype II ASFV group (1/3 pigs with mild severity). In non-lymphoid organs, renal tubular degeneration was detected only in the rASFV I/II group (1/3 pigs), while absent in genotype II ASFV-infected pigs. Hepatocellular degeneration was observed with similar frequency and severity in both groups (2/3 pigs with moderate severity). Pulmonary inflammatory cell infiltration showed variable patterns in both groups without clear differences.

**Table 2.** Comparative histopathological lesions at early viral infection (3 dpi) in spleen, gastrohepatic lymph node, lung, liver, and kidney from pigs experimentally infected with genotype II ASFV (*n* = 3) and rASFV I/II strains (*n* = 3).

		Histopathological lesion score					
		Genotype II ASFV			rASFV I/II		
Organ	Histopathological findings	#27	#39	#40	#18	#23	#25
Spleen	Congestion/hemorrhage	+++	+++	+++	+++	++	+++
	Lymphoid depletion	++	+	++	++	+	++
	Necrosis/Karyorrhexis	–	–	+	++	–	+
	Erythrophagocytosis	+++	+++	+++	+++	++	+++
	Endothelial injury/microthrombi	+	–	–	+	+	+
Submandibular lymph node	Congestion/hemorrhage	–	–	–	+	–	–
	Lymphoid depletion	++	+++	++	+	+	+++
	Necrosis/Karyorrhexis	–	–	+	++	+	++
	Erythrophagocytosis	–	++	+	+	+	++
	Endothelial injury/microthrombi	–	–	–	–	–	+
Lungs	Congestion/hemorrhage	–	–	+	+	+	–
	Alveolar edema	–	–	–	–	–	–
	Intra-alveolar fibrin	+	+	–	+	–	+
	Inflammatory cell infiltration	+++	+++	–	++	–	+++
	Endothelial injury/microthrombi	+	+	+	++	+	+
Liver	Congestion/hemorrhage	+	++	++	++	+	+
	Erythrophagocytosis	+	+	–	+	+	+
	Inflammatory cell infiltration	++	++	++	+	++	++
	Hepatocellular degeneration	–	++	++	++	–	++
	Endothelial injury/microthrombi	++	+	+	++	+	+
Kidneys	Congestion/hemorrhage	+	+	+	++	+	+
	Interstitial nephritis/edema	+	–	+	+	+	–
	Pigmented casts (heme/bile)	+	+	–	+	–	+
	Tubular degeneration	–	–	–	+	–	–
	Tubular necrosis	–	–	–	–	–	–

Scored according to our previously published guideline [20] with minor modifications. No lesion (–), mild (+), moderate (++), severe (+++).

## Discussion

Given the global unavailability of an effective ASF vaccine, understanding the pathogenicity of various ASFV variant strains is critical for disease control and the prevention of ASF outbreaks. This information is essential for guiding surveillance strategies, developing mitigation measures, and tailoring response plans to address the unique characteristics of emerging ASFV variant strains. To our knowledge, only a limited number of studies have comprehensively evaluated key clinical parameters – such as incubation period, clinical signs, and viremia – in pigs infected with rASFV I/II strains. These evaluations are particularly relevant as emerging strains may differ in pathogenicity, although further confirmation is needed.

The phylogenetic analysis of ASFV used in this study (ASF/HD 231005) revealed a genetically mosaic viral architecture, indicative of inter-genotypic recombination between genotype I and II ASFV. The *B646L* (p72) gene clearly classified this virus as genotype I, while the *E183L* (p54) gene exhibited high sequence similarity to genotype II ASFV, including strains from Georgia, China, and South Korea. Furthermore, the *EP402R* (CD2v) gene clustered within serogroup 8 (SG8), closely aligning with rASFV I/II strains previously reported in China and Inner Mongolia. In our previous study, genotype II ASFV caused death at 7.0 ± 1.2 dpi, whereas rASFV I/II resulted in a shorter time to death (5.5 ± 0.7 dpi). The onset of high fever appeared earlier (3 dpi), and the incubation period was also shorter (3.0 ± 1.1 dpi) in rASFV I/II-infected pigs compared to genotype II ASFV (3 dpi and 3.7 ± 0.5 dpi, respectively) [14]. Although these data were not collected in a strict side-by-side experiment, both studies were conducted under the same laboratory conditions – using identical facilities, staff, and diagnostic protocols – thereby minimizing inter-study variability and providing a reasonable basis for comparing the observed differences in disease progression. The shortened incubation period suggested the possibility that rASFV I/II strains may have distinct pathogenic characteristics with genotype II ASFV. Moreover, more than half of the infected pigs (*n* = 6, 60%) succumbed to the infection at 5 dpi, compared to only one pig (10%) inoculated with genotype II ASFV in our previous study. These findings also implied that rASFV I/II strains represent an emerging challenge in terms of disease severity and lethality compared to genotype II ASFV. The results of this study were in agreement with previous research conducted in China, which reported that rASFV I/II strains exhibit greater virulence than genotype II ASFV. These results highlighted the need for enhanced surveillance, rapid diagnostics, and the development of effective vaccines tailored to counter the emerging rASFV I/II strains. In their study [12], pigs infected with the rASFV I/II strains from China exhibited high fever onset at approximately 4 dpi and died within a range of 6 to 15 dpi. In contrast, our study demonstrated an earlier onset of high fever (2.6 ± 0.8 dpi) and a more compressed time-to-death range (5–7 dpi). We hypothesize that this discrepancy may be attributable to differences in the in vivo experimental conditions. Firstly, our study utilized domestic pigs free from major endemic diseases in Vietnam, whereas Zhao’s study [12] employed 7-week-old SPF pigs housed in BSL-3/ABSL-3 biosafety facilities. The pigs in the Chinese study were likely raised under more controlled conditions and were less affected by potential co-infections, which may have contributed to their slightly longer survival compared to the pigs in our study. A recent study also demonstrated that SPF status can influence ASFV disease progression, with SPF pigs often showing more predictable clinical outcomes and potentially extended survival times [21]. Secondly, while the Vietnamese rASFV I/II strains shared 99.86–99.98% nucleotide similarity with the Chinese strains, 39 distinct variations were identified in the Vietnamese isolates, distinguishing them from the Chinese rASFV I/II strains [17]. Previous studies suggest that these genetic differences may influence the virus’s pathogenicity and transmissibility [17]. It is reasonable to hypothesize that these mutations could contribute to the heightened virulence and accelerated mortality rates observed in our study. Specifically, variations in genes such as C122R, NP1450L, and I329L, which are associated with viral replication and immune response modulation, may enhance the virus’s ability to evade host defenses or improve replication efficiency. These genetic variations could explain the distinct pathogenic profile of the Vietnamese rASFV I/II strains.

The narrower time window for disease progression observed in our study may have significant implications for detection and intervention strategies, emphasizing the urgency of rapid diagnostics and immediate responses. These measures will be critical for guiding biosecurity protocols and preparedness strategies, especially in the absence of an effective vaccine. Previously, we suggested that rope-based oral fluid sampling is one of the most reliable methods for early detection in genotype II ASFV-infected pigs and those exposed through contact [14,15]. Consistent with these findings, viral genome copies were detected as early as 2 dpi in the rASFV I/II-infected herd (rVI group), preceding the mean incubation period (3.0 ± 1.1 dpi) for rASFV I/II. The viral excretion patterns observed in individual samples (blood, nasal swabs, oral swabs, and rectal swabs) from the rVI group pigs were similar to those reported in previous studies on genotype II ASFV-infected pigs [14]. However, viral DNA was detected more rapidly in herd-based samples from the rVI group than in those from the genotype II ASFV-infected group, including rope-based oral fluids, pooled feces on the floor, water, and feed. These findings implied that rASFV I/II strains may necessitate more rigorous control measures compared to genotype II ASFV, given their enhanced possibility to cause severe outbreaks and rapid mortality. Future studies should aim to elucidate the genetic or molecular factors driving the pathogenicity of rASFV I/II strains, as well as their transmissibility and environmental stability.

Postmortem examination results indicated that the rVI group pigs exhibited gross lesions similar to those observed in our previous studies on genotype II ASFV-infected pigs [14,15,20]. Hallmarks from the acute form of ASFV infection in pigs, including congestive splenomegaly, congestive lymphadenopathy in various lymph nodes, and hemorrhagic lesions in vital organs, were evident in rASFV I/II-infected pigs. However, pigs in the rVI group in our study displayed slightly more pronounced hemorrhagic lesions in the lungs and kidneys compared to genotype II ASFV-infected pigs in our previous study [20], suggesting that rASFV I/II exhibits a different pattern of vascular damage with genotype II ASFV. In addition, the rVI group pigs had significantly higher viral genome copy numbers in the lungs (rASFV I/II: 7.34 ± 0.44; genotype II: 5.46 ± 5.30) and kidneys (rASFV I/II: 6.67 ± 0.42; genotype II: 4.80 ± 4.84) compared to genotype II ASFV-infected pigs [14]. In the kidneys, our previous studies reported that genotype II ASFV-infected pigs showed more severe renal lesions during the later stages of infection [20]. Moreover, our previous study already revealed that ASFV was spreading rapidly in the spleen of pig body following viremia, and then broadly disseminated to other organs as follows: the liver, lungs, SLN, MLN, and kidneys [22]. Therefore, the high viral genome copy numbers in the kidneys of rVI group pigs suggest that rASFV I/II may have a greater propensity for rapid dissemination within the host compared to genotype II ASFV.

Moreover, although the rVI group pigs in this study succumbed earlier than genotype II ASFV-infected pigs in previous studies, the average viral genome copy numbers across all collected organs from rASFV I/II-infected pigs were higher than those from genotype II ASFV (except for the GLN, brain, and spinal cord, which were not tested in our previous studies) [14]. These findings support the hypothesis that rASFV I/II strains could be more virulent and replicate more efficiently in the host than genotype II ASFV. To partially address this, we conducted a preliminary comparative study investigating histopathological lesions at 3 dpi between genotype II ASFV and rASFV I/II-infected pigs. Although the sample size was limited (*n* = 3 per group) and observed only at a single early time point, these findings demonstrated distinct pathological features of rASFV I/II infection. Notably, the lesions of endothelial injury with fibrin microthrombi formation were observed in all rASFV I/II-infected pigs, while only one genotype II ASFV-infected pig showed this lesion. Moreover, rASFV I/II infection showed different patterns of tissue lesions, including more frequent lymphoid organ necrosis and presentation of renal tubular degeneration. These early pathological differences may contribute to the accelerated disease progression in rASFV I/II infection.

Given that the gross lesions in rVI group pigs were similar with to those observed in genotype II ASFV-infected pigs, it is challenging to differentiate whether ASF-suspected pigs were infected with rASFV I/II or genotype II ASFV based solely on postmortem examinations. However, one distinctive gross lesion was identified in rASFV I/II-infected pigs: jaundice. Jaundice was observed in all rVI group pigs, with 90% exhibiting severe jaundice. Since the livers of rVI group pigs appeared grossly intact or showed no significant lesions, the hemolytic jaundice observed may be attributable to severe and rapid red blood cell destruction caused by rASFV I/II infection, which likely induces severe hemorrhagic injury during the very early stages of infection. Moreover, the severe splenic congestion and abundance of hemosiderin-laden macrophages observed in histopathological analysis, which are hallmark indicators of extensive erythrocyte destruction and hemoglobin breakdown. This massive hemolysis would release large quantities of bilirubin that overwhelmed the clearance capacity of the liver, leading to hyperbilirubinemia and icterus. In the liver, severe congestion without hepatocellular necrosis was observed, indicating that hepatic function was almost intact. These histopathological findings strongly supported that the gross lesion of jaundice was not derived from hepatocyte failure or cholestasis, but from excess bilirubin load due to severe congestion. Notably, jaundice has not been a prominent clinical gross lesion of genotype II ASFV infection. In contrast, these results suggested that rASFV I/II infection maybe trigger severe hemolysis – manifesting as hemolytic anemia – in pigs, which could result in jaundice.

In China, genotype II ASFV was first introduced in 2018 and circulated for over two years. By 2020, a less virulent genotype II ASFV variant was detected, likely due to natural mutations in the highly virulent virus [23]. In 2021, a low-virulence genotype I ASFV strain was reported on pig farms in Henan and Shandong provinces, resembling ASFV strains isolated in Portugal during the 1960s and 1980s [24]. In contrast, Vietnam has conducted limited surveillance studies to monitor different ASFV strains [25,26]. Two previous studies identified novel variants of the p72 genotype II ASFV. Given Vietnam’s geographical proximity to China, it is plausible that new or diverse ASFV strains are emerging and circulating within the country. Currently, two live-attenuated ASFV vaccines have been approved in Vietnam, demonstrating protective efficacy against circulating genotype II ASFV. Both vaccines are recommended for pigs aged 4 weeks or older, with a single dose providing protection against ASF p72 genotype II viruses currently circulating in Vietnam [27,28]. However, recent studies have indicated that the rASFV I/II strain and low-virulence genotype I ASFV isolates were not effectively neutralized by live-attenuated genotype II ASFV candidate vaccines in China [12,29]. Consequently, the emergence of rASFV I/II strains emphasizes the urgent need to develop or refine vaccine platforms that can confer cross-genotype protection, particularly given recent evidence suggesting limited efficacy of existing genotype II ASFV-based candidates against these recombinant variants.

In conclusion, our study revealed that the pathogenicity of emerging rASFV I/II strain from Vietnam in domestic pigs, demonstrated distinct pathobiological characteristics that differ from genotype II ASFV. The rASFV I/II-infected pigs presented a shorter incubation period, earlier onset of high fever, rapid progression to death, and higher viral genome copy numbers in various organs, along with more severe hemorrhagic lesions, particularly in the lungs and kidneys. These findings implied the potentially significant threat posed by rASFV I/II to the swine industry and highlight the urgent need for enhanced surveillance, rapid diagnostic methods, and the development of effective vaccines capable of targeting multiple ASFV variants. Further studies are necessary to elucidate the molecular mechanisms underlying the increased virulence of rASFV I/II, which will be critical for developing effective control and prevention strategies against this emerging variant.

## Supplementary Material

Figure S1.jpg

Figure S2.jpg

Figure S1 supplementary_re_revised_0711.docx

Figure S2 supplementary.docx

## Data Availability

The data that support the findings of this study are openly available in FigShare at “https://doi.org/10.6084/m9.figshare.28190330.v1.” The whole-genome sequence, *B646L*, *E183L*, and *EP402R* genes of the 231005 strain used in this study are available in GenBank of the National Center for Biotechnology Information (https://www.ncbi.nlm.nih.gov/genbank/) under accession numbers PX212717 (https://www.ncbi.nlm.nih.gov/nuccore/PX212717), PQ328515 (https://www.ncbi.nlm.nih.gov/nuccore/PQ328515), PV614732 (https://www.ncbi.nlm.nih.gov/nuccore/PV614732), and PV614731 (https://www.ncbi.nlm.nih.gov/nuccore/PV614731), respectively.
