# Supplementary figures and images for: Pathogenicity and pathobiological characterization of a recombinant genotype I/II African swine fever virus in pigs
Source: Virulence. 2025 Oct 25;16(1):2580123. doi: 10.1080/21505594.2025.2580123 (PMC12562799; doi:10.1080/21505594.2025.2580123)

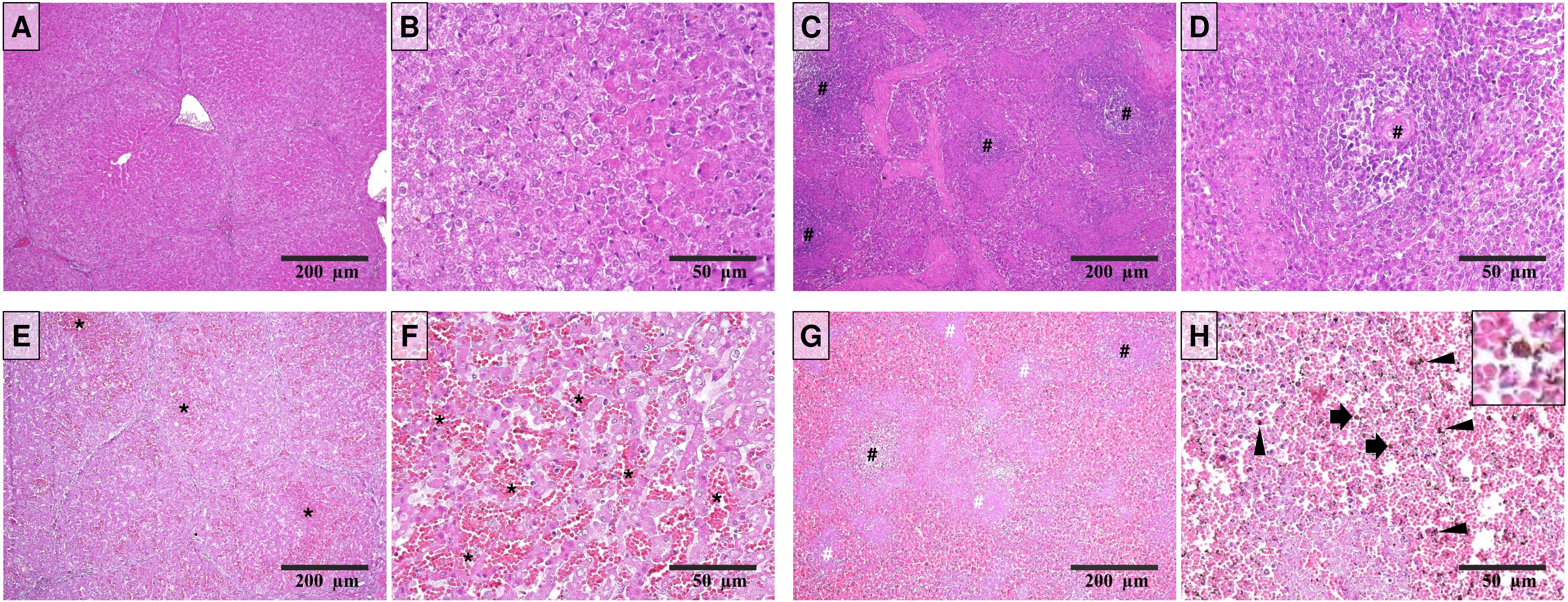

Supplement: Figure S1.jpg [file KVIR_A_2580123_SM1600.jpg]

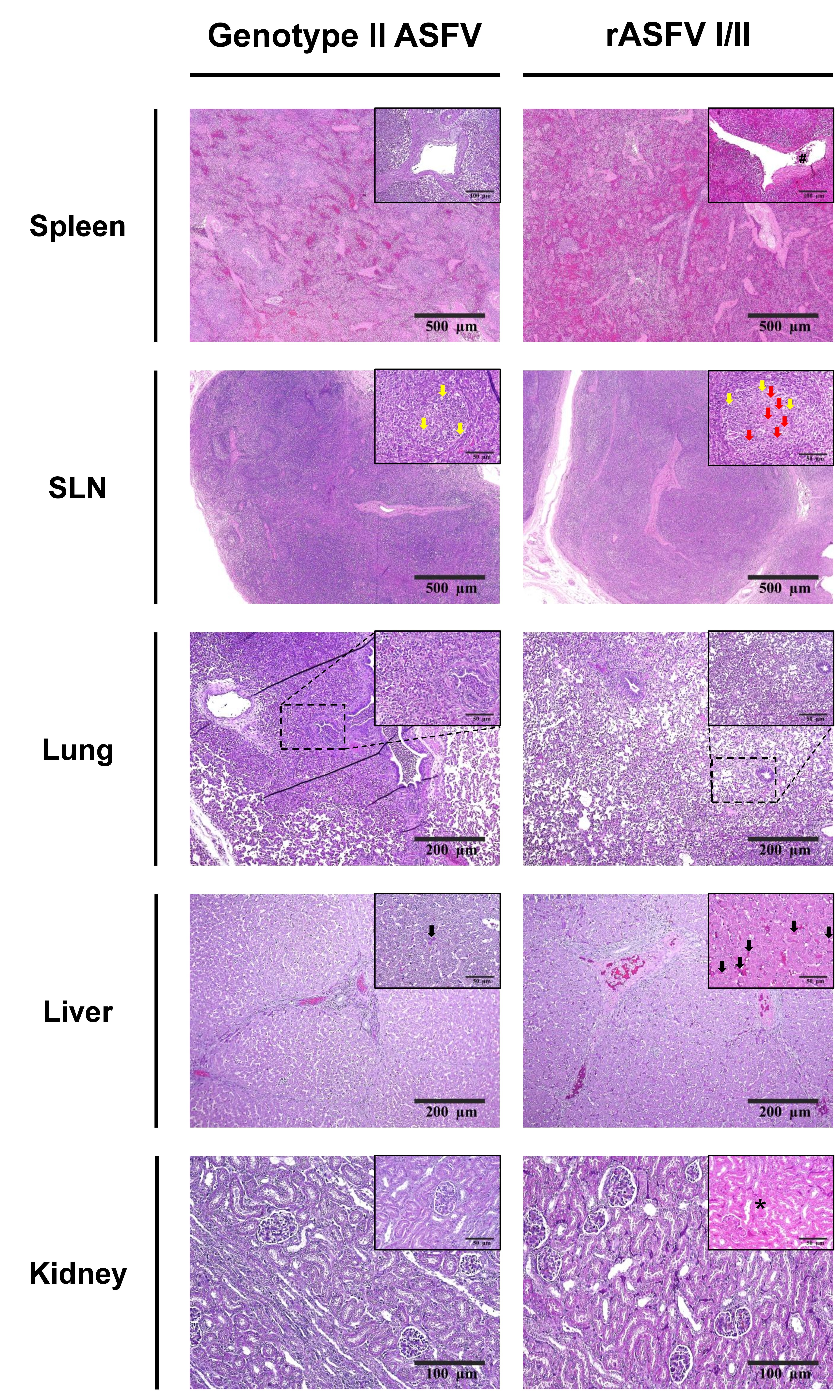

Supplement: Figure S2.jpg [file KVIR_A_2580123_SM1599.jpg]
