# Supplementary material for: Pathogenicity and pathobiological characterization of a recombinant genotype I/II African swine fever virus in pigs
Source: Virulence. 2025 Oct 25;16(1):2580123. doi: 10.1080/21505594.2025.2580123 (PMC12562799; doi:10.1080/21505594.2025.2580123)
Supplement: Figure S1 supplementary_re_revised_0711.docx [file KVIR_A_2580123_SM1598.docx]

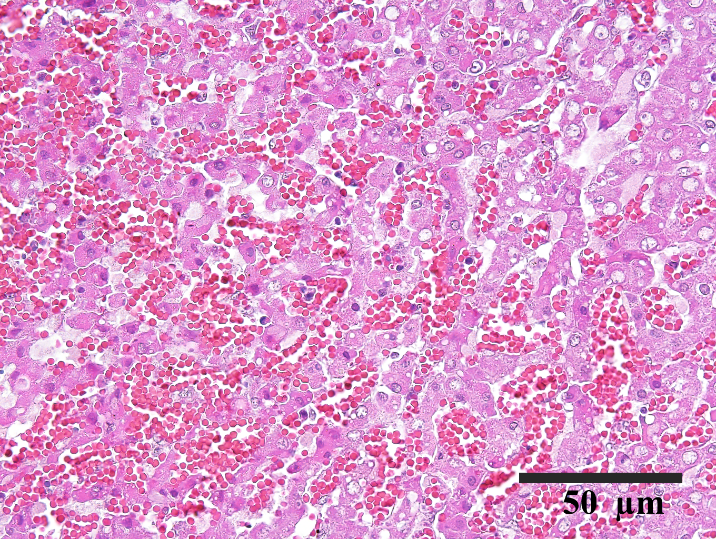

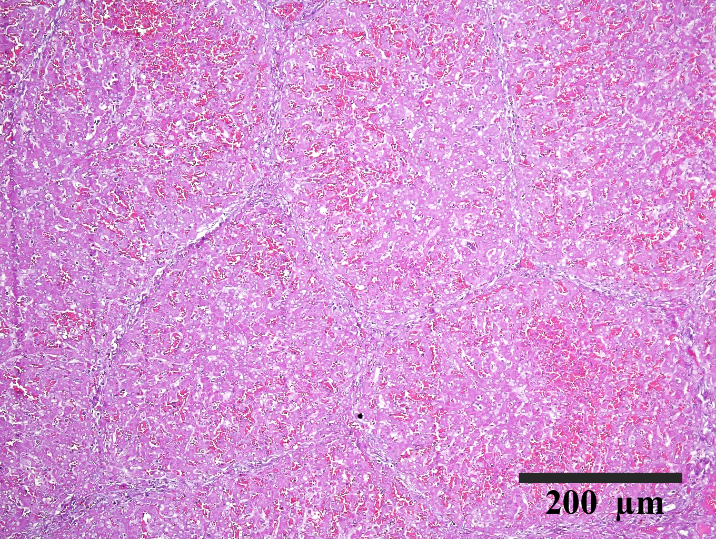

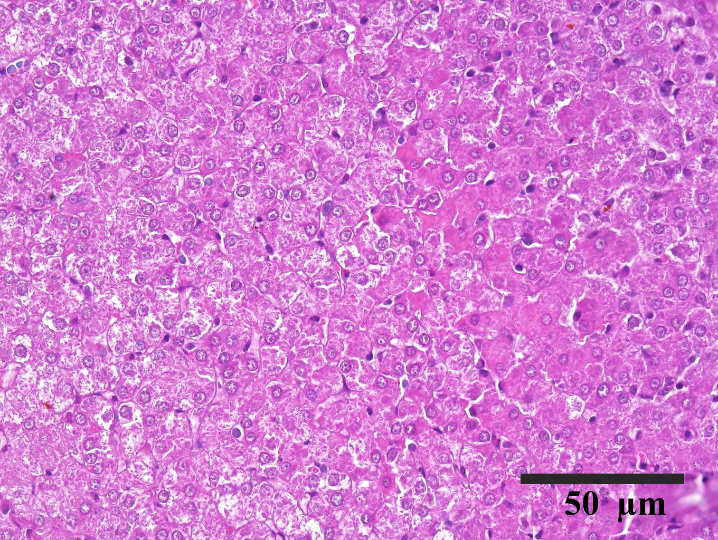

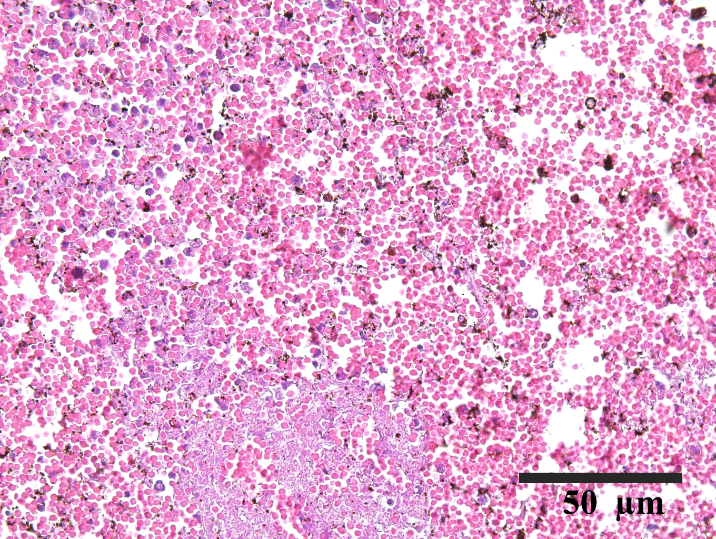

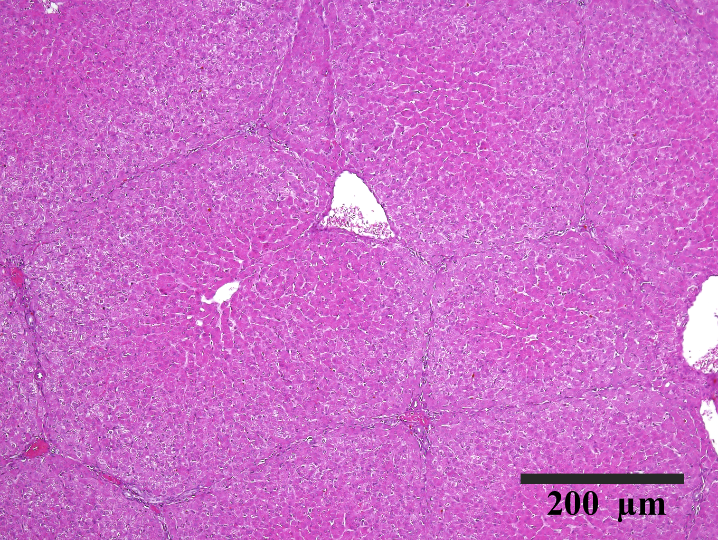

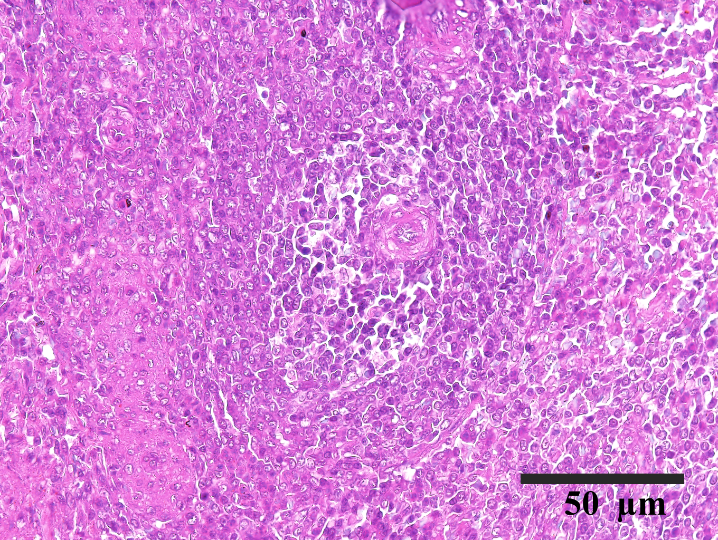

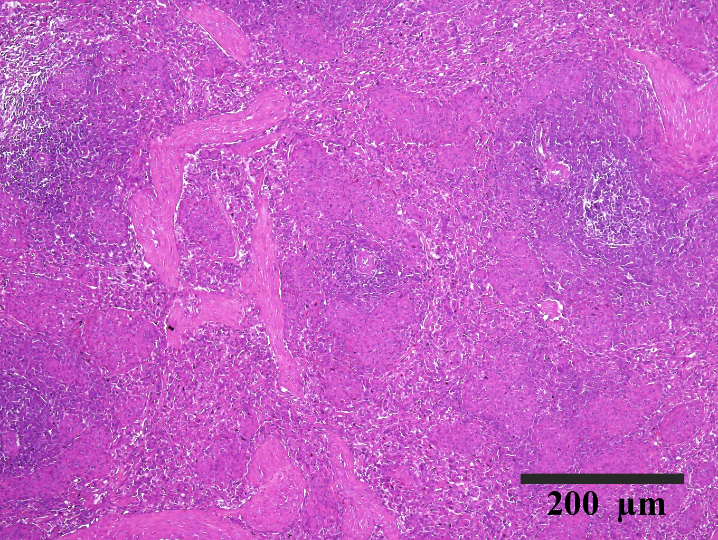

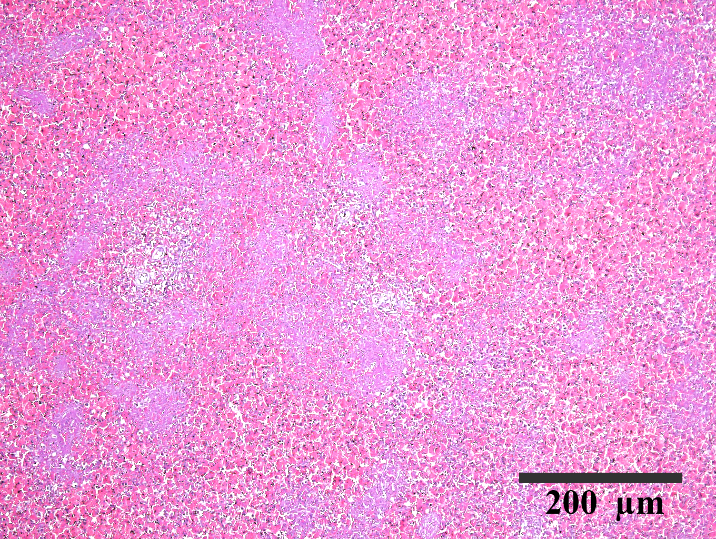

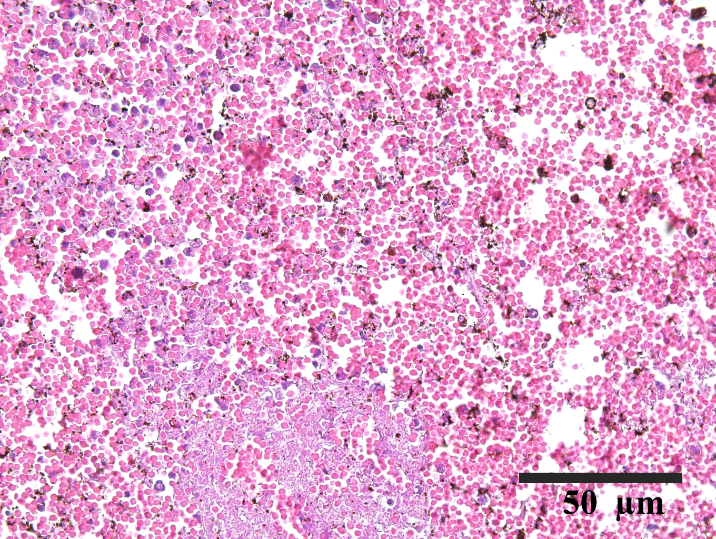


**D**

**C**

**B**

**A**

*****

*****

*****

*****

*****

*****

*****

*****

*****

**#**

**#**

**#**

**#**

**#**

**#**

**#**

**#**

**#**

**#**

**#**

**H**

**G**

**F**

**E**

**Figure S1. Representative histopathological lesions of liver and spleen in rASFV I/II-infected pigs and negative control pigs. (A, B)** Liver. Negative control group pigs. **(C, D)** Spleen. Negative control group pigs. White pulp is indicated by black crosshatches. **(E, F)** Liver. rASFV I/II-infected pigs. Severe congestion. Congested areas are marked with asterisks. **(G, H)** Spleen. rASFV I/II-infected pigs. Severe congestion. Atrophy of white pulp (black crosshatch) and necrotic lesions (white crosshatch) were observed. **(H)** Numerous hemosiderins (arrowheads) and hemosiderin-laden macrophages (arrows; inset) were observed. H&E stain.
