# Supplementary material for: Pathogenicity and pathobiological characterization of a recombinant genotype I/II African swine fever virus in pigs
Source: Virulence. 2025 Oct 25;16(1):2580123. doi: 10.1080/21505594.2025.2580123 (PMC12562799; doi:10.1080/21505594.2025.2580123)
Supplement: Figure S2 supplementary.docx [file KVIR_A_2580123_SM1597.docx]

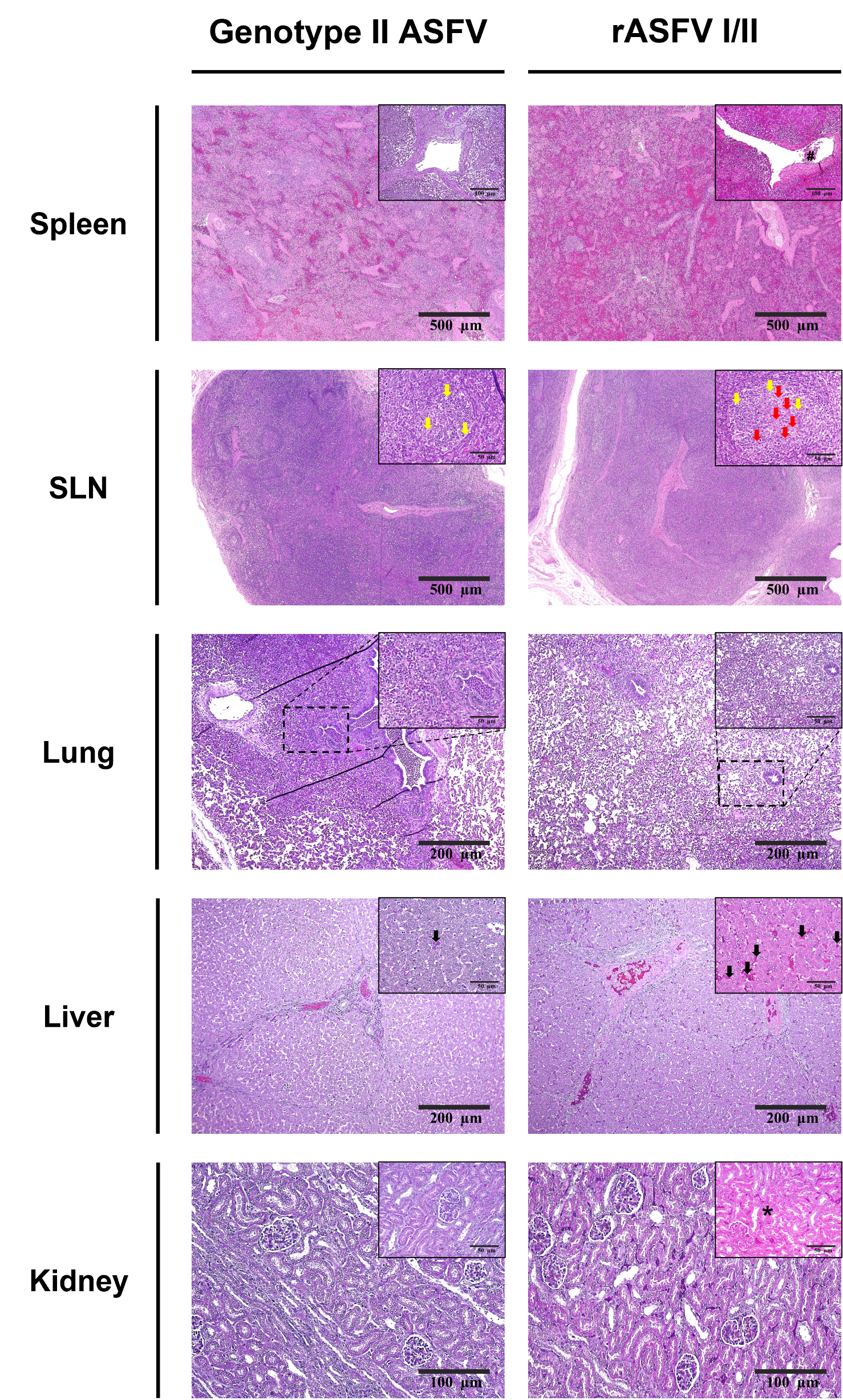


**Figure S2. Representative histopathological lesions from five major organs in pigs inoculated with genotype II ASFV and rASFV I/II at 3 days post-inoculation.** In spleen, both groups showed congestion and hemorrhage. Note the presence of endothelial injury and microthrombi formation (black crosshatch) in rASFV I/II group. In submandibular lymph node (SLN), lymphoid depletion was observed in both groups, and moderate lesions of necrosis (red arrows) and karyorrhexis (yellow arrows) in rASFV I/II group. In lung section, variable inflammatory cell infiltration patterns are observed in both groups. In liver, both groups displayed similar degrees of congestion and hepatocellular degeneration (black arrows) without significant necrosis. In kidney, the renal tubular structures appeared relatively preserved in both groups at this early time point, although tubular degeneration (asterisk) was observed in one rASFV I/II-infected pig. All sections were stained with hematoxylin and eosin. Magnification: 40× (Spleen, SLN), 100× (Lung, Liver), 200× (Kidney, Insert - Spleen) and 400× (Inset - SLN, Lung, Liver, Kidney).
